# Analysis of Genetic Variation and Enhancement of Salt Tolerance in French Pea (*Pisum Sativum* L.)

**DOI:** 10.3390/ijms19082433

**Published:** 2018-08-17

**Authors:** Mohamed A. El-Esawi, Abdullah A. Al-Ghamdi, Hayssam M. Ali, Aisha A. Alayafi, Jacques Witczak, Margaret Ahmad

**Affiliations:** 1 Botany Department, Faculty of Science, Tanta University, Tanta 31527, Egypt; 2 UMR CNRS 8256 (B2A), IBPS, Université Paris VI, 75005 Paris, France; jacques.witczak@upmc.fr (J.W.); margaret.ahmad@upmc.fr (M.A.); 3 Botany and Microbiology Department, College of Science, King Saud University, P.O. Box 2455, Riyadh 11451, Saudi Arabia; aaalgham@yahoo.com (A.A.A.-G.); hayhassan@ksu.edu.sa (H.M.A.); 4 Timber Trees Research Department, Sabahia Horticulture Research Station, Horticulture Research Institute, Agriculture Research Center, Alexandria 21526, Egypt; 5 Biological Sciences Department, Faculty of Science, University of Jeddah, Jeddah 21577, Saudi Arabia; aal_shareaf@hotmail.com; 6 Department of Biology, Xavier University, Cincinnati, OH 45207, USA

**Keywords:** pea, fatty acid, AFLP diversity, marker-trait association, salt tolerance

## Abstract

*Pisum sativum* L. (field pea) is a crop of a high nutritional value and seed oil content. The characterization of pea germplasm is important to improve yield and quality. This study aimed at using fatty acid profiling and amplified fragment length polymorphism (AFLP) markers to evaluate the variation and relationships of 25 accessions of French pea. It also aimed to conduct a marker-trait associations analysis using the crude oil content as the target trait for this analysis, and to investigate whether 5-aminolevulinic acid (ALA) could enhance salt tolerance in the pea germplasm. The percentage of crude oil of the 25 pea genotypes varied from 2.6 to 3.5%, with a mean of 3.04%. Major fatty acids in all of the accessions were linoleic acid. Moreover, the 12 AFLP markers used were polymorphic. The cluster analysis based on fatty acids data or AFLP data divided the 25 pea germplasm into two main clusters. The gene diversity of the AFLP markers varied from 0.21 to 0.58, with a mean of 0.41. Polymorphic information content (PIC) of pea germplasm varied from 0.184 to 0.416 with a mean of 0.321, and their expected heterozygosity (*H_e_*) varied from 0.212 to 0.477 with a mean of 0.362. The AFLP results revealed that the Nain Ordinaire cultivar has the highest level of genetic variability, whereas Elatius 3 has the lowest level. Three AFLP markers (E-AAC/M-CAA, E-AAC/M-CAC, and E-ACA/M-CAG) were significantly associated with the crude oil content trait. The response of the Nain Ordinaire and Elatius 3 cultivars to high salinity stress was studied. High salinity (150 mM NaCl) slightly reduced the photosynthetic pigments contents in Nain Ordinaire leaves at a non-significant level, however, the pigments contents in the Elatius 3 leaves were significantly reduced by high salinity. Antioxidant enzymes (APX—ascorbate peroxidase; CAT—catalase; and POD—peroxidase) activities were significantly induced in the Nain Ordinaire cultivar, but non-significantly induced in Elatius 3 by high salinity. Priming the salt-stressed Nain Ordinaire and Elatius 3 plants with ALA significantly enhanced the pigments biosynthesis, antioxidant enzymes activities, and stress-related genes expression, as compared to the plants stressed with salt alone. In conclusion, this study is amongst the first investigations that conducted marker-trait associations in pea, and revealed a sort of correlation between the diversity level and salt tolerance.

## 1. Introduction

Field pea (*Pisum sativum* L.) has a high nutritional value and seed oil content [1]. In addition, due to its high ability to fix nitrogen, pea could be efficiently used as a biodiesel resource to replace canola oil and other oilseed crops that require high costs for use in biodiesel production [2]. With respect to productivity, pea ranks fourth worldwide after soybean, peanut, and dry bean [3]. Although pea germplasm is rich in genetic diversity, a few research studies have been conducted and have shown a low oil content in peas varying between 1.5% and 3.7% [3,4,5], indicating that the currently available cultivars have a limited oil content. The oil content in pea is also low compared with that of canola and soybean [6]. As a result, effective breeding programmes should be conducted to develop improved pea genotypes with a promising oil content, which could be efficiently used for biodiesel production or as a dietary source. Furthermore, investigating fatty acid profiling and abiotic stress tolerance in crop varieties is important and would provide useful information about the best candidate varieties, which could be selected and used in breeding programs as well as in the classification of newly developed or uncharacterized varieties maintained at gene banks [6].

The breeders have reported a further need for harnessing genetic diversity for crop improvement, particularly for self-pollinating crops such as *Pisum sativum* L. [2,7]. Several methodologies have been utilized to assess the genetic relatedness and physiological processes in plants, including morphological, biochemical, and molecular markers [8,9,10,11,12,13,14,15,16,17,18,19,20,21,22,23,24,25,26,27]. Molecular markers are efficient in the evaluation of variation and phylogenetic studies. Amplified fragment length polymorphism (AFLP) proved useful to investigate the diversity levels and relationships in pea germplasm, because of their high degree of polymorphism [2,28,29,30,31,32,33,34,35]. Smýkal et al. [32] used microsatellites to evaluate the variability and structure of 164 pea accessions, and reported a considerable level of diversity among accessions. Deulvot et al. [34] used single nucleotide polymorphisms to perform a pea genetic map with 37 new makers. Ahmad et al. [2] also used microsatellites to study the genetic structure and marker-trait associations in *Pisum sativum* L., and reported some valuable pea varieties to be used as parental candidates in breeding programs. Furthermore, Simioniuc et al. [30] used AFLPs to assess the variability and phylogenetic relationships of 21 pea varieties released in Germany. Dyachenko et al. [35] also successfully evaluated the levels of variation among different pea varieties using AFLP markers.

The present study aimed at using fatty acid profiling and AFLP markers to assess the variation and relationships of 25 varieties of French pea deposited at the Netherlands Centre for Genetic Resources. This study also aimed at conducting a marker-trait associations analysis using the crude oil content as the target trait for this analysis. Additionally, this study investigated the correlation between the genetic variability level and the salt tolerance degree in two genotypes comprising the highest and lowest genetic variability levels among all of the genotypes studied, and also investigated whether the growth regulator 5-aminolevulinic acid (ALA) could further improve the salt tolerance in these two genotypes.

## 2. Results and Discussion

### 2.1. Crude Oil Content and Fatty Acid Analysis

**Figure 1:**
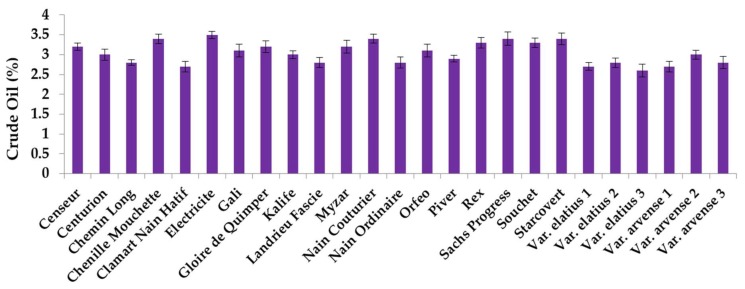
Percentage of crude oil content in the 25 accessions of *Pisum sativum* L.

The crude oil content and fatty acid analysis of each of the 25 accessions of *Pisum sativum* L. have been studied. Figure 1 shows the percentage of crude oil content in each of the 25 accessions. This percentage of crude oil content ranged from 2.6 (Elatius 3) to 3.5% (Electricite genotype), with an average of 3.04%. These values were relatively close to that reported by Murcia and Rincon [36,37].

**Figure 2:**
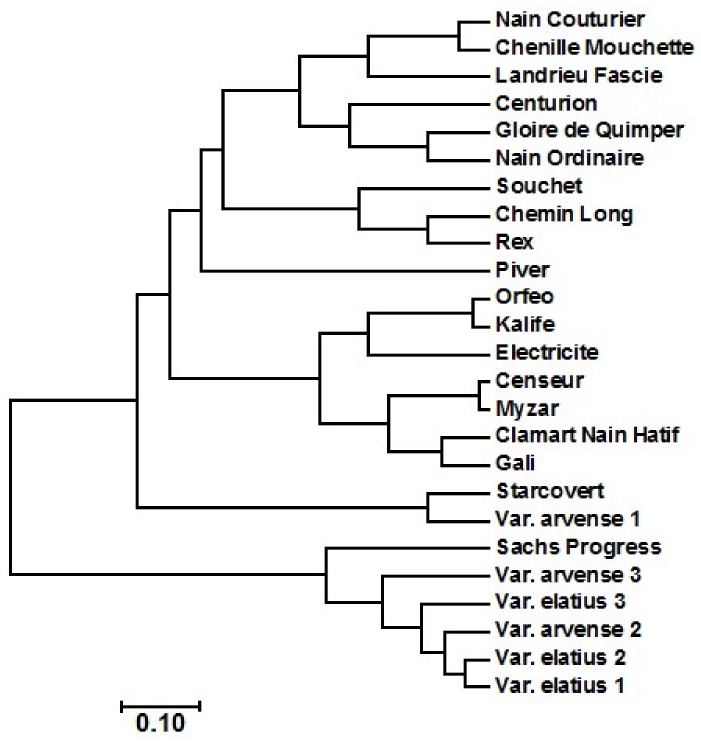
Cluster analysis of the 25 accessions of *Pisum sativum* L. using fatty acids data and crude oil content.

Table 1 shows the composition of fatty acid of the 25 accessions of *Pisum sativum* L. All of the accessions comprised palmitic (13.6 to 18.4), stearic (13.1 to 16.2), oleic (18.7 to 25.6), linoleic (24.8 to 35.2), and linolenic (11 to 14.2). The results indicated that the main fatty acid in the pea varieties was linoleic acid. These results agreed with that recorded by Murcia and Rincon [36,37]. However, the values of the fatty acids of the present study are variable, compared to that recorded in the previous studies [4,5,36,37]. The cluster analysis of the 25 accessions of *Pisum sativum* L. was conducted based on the fatty acids data and crude oil content (Figure 2), and divided the 25 accessions into 2 major clusters. The first cluster contained varieties of Sachs Progress, Elatius 1, Elatius 2, Elatius 3, Arvense 2, and Arvense 3, while the second cluster contained the remaining 19 accessions.

### 2.2. Amplified Fragment Length Polymorphism (AFLP) Diversity Analysis

Twelve AFLP primer pairs were utilized to evaluate the variability of the 25 pea accessions (Table 2). Number of AFLP bands identified for each primer set ranged from 29 to 89, with a mean of 55.75 (Table 2). The primer set (E-AAT/M-CAT) exhibited the lowest number of amplified AFLP fragments (29) (Table 2). The primer pair (E-ACA/M-CTG) indicated the highest number of amplified AFLP fragments (89). All of the 12 primer pairs were polymorphic. The number of polymorphic bands for each AFLP primer set ranged from 15 (E-AAT/M-CAT) to 54 (E-ACA/M-CGA), with a mean of 35.25. Moreover, the polymorphism percentage ranged from 51.2% (E-ACA/M-CAG) to 80.4% (E-ACA/M-CAC), with a mean of 62.83% (Table 2). The gene diversity varied from 0.21 (E-ATG/M-CAG) to 0.58 (E-ACA/M-CTG), with a mean of 0.41, suggesting a considerable level of polymorphism. The polymorphic information content (PIC) varied from 0.16 (E-ATG/M-CAG) to 0.54 (E-ACA/M-CTG), with a mean of 0.37 (Table 2). Table 3 reveals the diversity indices estimated for each of the 25 genotypes of *Pisum sativum* L. and wild accessions. The observed heterozygosity (*H*_o_) varied from 0.197 (Chenille Mouchette) to 0.322 (Arvense 2), with a mean of 0.273. The expected heterozygosity (*H*_e_) ranged from 0.212 (Elatius 3) to 0.477 (Nain Ordinaire), with a mean of 0.362. The PIC varied from 0.184 (Elatius 3) to 0.416 (Nain Ordinaire), with a mean of 0.321, indicating that the varieties comprised a considerable level of variability, which was higher than the variability level recorded in the pea germplasm by Ahmad et al. [2]. This result reflects that the Nain Ordinaire accession has the highest level of genetic variability, but the Elatius 3 accession has the lowest variability level. Four accessions (Rex, Starcovert, Elatius 2, and Elatius 3) also revealed negative fixation indices, indicating a higher level of heterozygosity in those four accessions (Table 3). These four accessions could particularly be exploited in future breeding strategies, in order to broaden the pea genetic base and improve the crop productivity and quality. 

**Figure 3:**
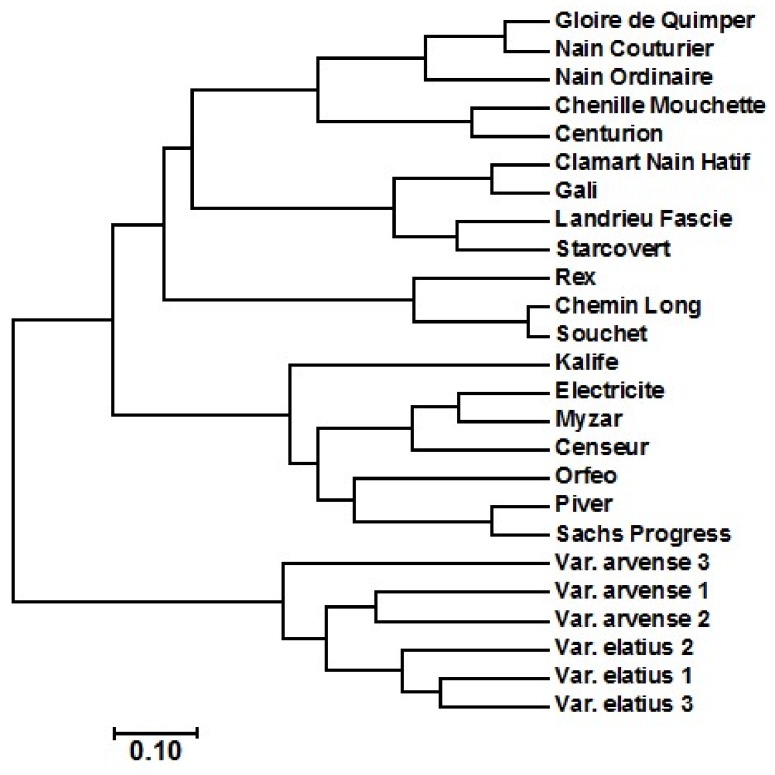
Cluster analysis of the 25 accessions of *Pisum sativum* L. using AFLP data.

Nei’s distance-based dendrogram connducted based on the AFLP data showed the relationships among the 25 pea genotypes analyzed (Figure 3). The dendrogram revealed two main clusters; one cluster contained Elatius 1, Elatius 2, Elatius 3, Arvense 1, Arvense 2, and Arvense 3, while the second cluster comprised all of the remaining genotypes. These results relatively agreed with the data of the cluster analysis based on fatty acids data, with some slight differences in the pea varieties grouping (i.e., in Figure 2, Sachs Progress variety was clustered with the varieties of Arvense 2, Arvense 3, Elatius 1, Elatius 2, and Elatius 3 of the first cluster, but Arvense 1 was clustered with the remaining varieties in the second cluster). 

### 2.3. Marker–Trait Association

A threshold of *p* = 0.05 was used to study and determine the marker–trait associations. A total of 12 combinations were evaluated to determine associations between AFLP traits and crude oil content based on a mixed linear model. Three AFLP markers, E-AAC/M-CAA, E-AAC/M-CAC, and E-ACA/M-CAG, of *p*-values 0.0011, 0.0272, and 0.0412, respectively, were significantly linked with the crude oil content trait. These three AFLP markers revealing a significant association with the crude oil content, could be exploited in breeding practices in order to enhance the crop quality and to develop new varieties of high yield. Ahmad et al. [2] also indicated that four microsatellite markers were significantly linked with the lipid content trait in *Pisum sativum* L. These markers could help in marker-assisted selection studies for improving and developing the cultivars of highly agronomic traits.

### 2.4. Salt Tolerance Test and the Effect of 5-Aminolevulinic Foliar Spray 

Our AFLP analysis results revealed that the Nain Ordinaire cultivar has the highest level of genetic variability, whereas the Elatius 3 cultivar has the lowest variability level. Therefore, we selected these two variable cultivars to study their response to salinity and investigate whether there is a sort of correlation between their genetic diversity level and their salt tolerance degree. Here, we also further investigated whether the growth regulator 5-aminolevulinic acid (ALA) could improve the salt tolerance in these two genotypes.

Table 4 shows that the high salinity (150 mM NaCl) slightly reduced the contents of chlorophyll and carotenoid pigments in the leaves of the Nain Ordinaire cultivar at a non-significant level, relative to the untreated control plants. Priming the salt-stressed Nain Ordinaire plants with 5-aminolevulinic acid (ALA) significantly improved the contents of all of the photosynthetic pigments, as compared to the plants stressed with salt alone (Table 4). Moreover, the treatment of the non-stressed Nain Ordinaire plants with ALA significantly enhanced the contents of the photosynthetic pigments, relative to the untreated control plants. Table 5 shows that high salinity significantly reduced the contents of all of the photosynthetic pigments in the leaves of the Elatius 3 cultivar, relative to the untreated control plants. Priming the salt-stressed Elatius 3 plants with ALA significantly enhanced the contents of all of the photosynthetic pigments, as compared to the plants stressed with salt alone (Table 5). Furthermore, the treatment of the non-stressed Elatius 3 plants with ALA significantly improved the photosynthetic pigments contents, relative to the untreated control plants.

**Figure 4:**
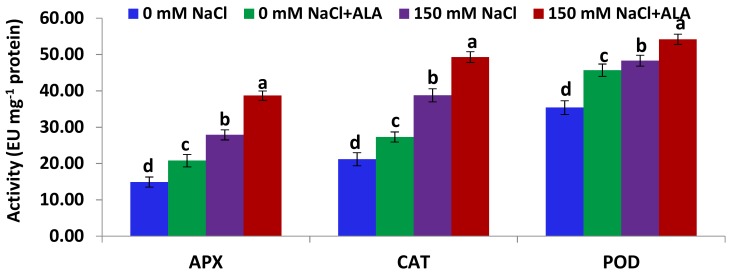
Activities of ascorbate peroxidase (APX), catalase (CAT), and peroxidase (POD) in the leaves of the Nain Ordinaire cultivar following salinity (150 mM NaCl) and 5-aminolevulinic acid ([ALA], 6 ppm) treatments. Data are means ± standard error (SE) (*n* = 4). Different letters indicate significant differences between treatments (*p* ≤ 0.05).

**Figure 5:**
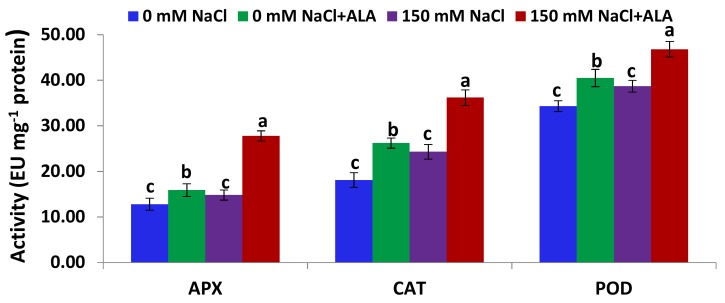
Activities of APX, CAT, and POD in the leaves of the Elatius 3 cultivar following salinity (150 mM NaCl) and 5-aminolevulinic acid ([ALA], 6 ppm) treatments. Data are means ± SE (*n* = 4). Different letters indicate significant differences between treatments (*p* ≤ 0.05).

Figure 4 shows that the activities of the three antioxidant enzymes (APX—ascorbate peroxidase; CAT—catalase; and POD—peroxidase) in the leaves of the Nain Ordinaire cultivar were significantly induced under saline conditions, relative to the untreated control plants. Treatment of the salt-stressed Nain Ordinaire plants with ALA also significantly induced the activities of the three enzymes, as compared with the plants stressed with salt alone (Figure 4). Furthermore, priming the non-stressed Nain Ordinaire plants with ALA significantly improved the three antioxidant enzymes’ activities, relative to the untreated control plants. Figure 5 shows that the antioxidant enzymes activities in leaves of the Elatius 3 cultivar were induced under salt-stress conditions, but at a non-significant level, relative to the untreated control plants. This induction level was lower than that recorded in the Nain Ordinaire leaves. Priming the salt-stressed Elatius 3 plants with ALA significantly induced the activities of the three enzymes, compared with the plants stressed with salt alone (Figure 5). Additionally, the treatment of the non-stressed Elatius 3 plants with ALA significantly enhanced the three antioxidant enzymes’ activities, relative to the untreated control plants.

**Figure 6:**
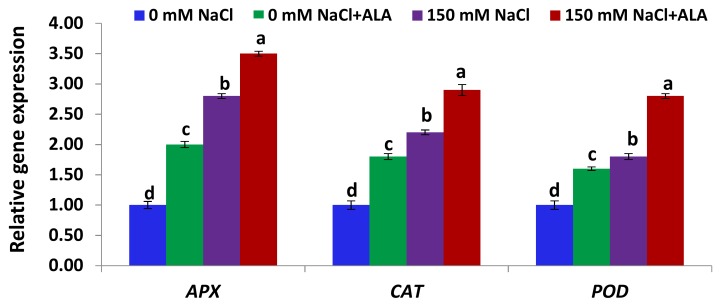
Expression levels of *APX*, *CAT*, and *POD* genes in leaves of Nain Ordinaire cultivar following salinity (150 mM NaCl) and 5-aminolevulinic acid ([ALA], 6 ppm) treatments. Data are means ± SE (*n* = 4). Different letters indicate significant differences between treatments (*p* ≤ 0.05).

**Figure 7:**
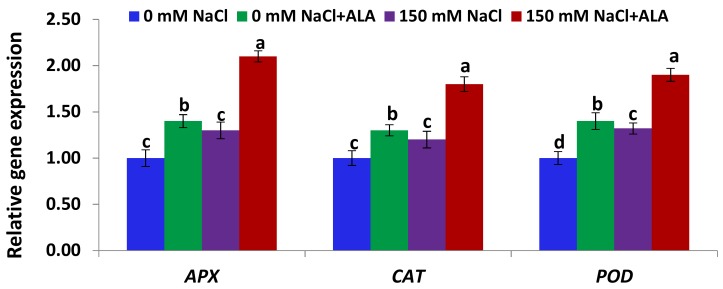
Expression levels of *APX*, *CAT*, and *POD* genes in leaves of the Elatius 3 cultivar following salinity (150 mM NaCl) and 5-aminolevulinic acid ([ALA], 6 ppm) treatments. Data are means ± SE (*n* = 4). Different letters indicate significant differences between treatments (*p* ≤ 0.05).

Figure 6 shows that the expression levels of the genes encoding the three antioxidant enzymes (APX, CAT, and POD) in the leaves of the Nain Ordinaire cultivar were significantly induced under salt-stress, relative to the untreated control plants. Priming the salt-stressed Nain Ordinaire plants with ALA also significantly induced the expression of the three antioxidant genes, compared to the plants stressed with salt alone (Figure 6). Additionally, treating the non-stressed Nain Ordinaire plants with ALA significantly induced the expression of the three antioxidant genes, relative to the untreated control plants. Figure 7 shows that the expression levels of the genes encoding the three antioxidant enzymes (APX, CAT, and POD) in the leaves of the Elatius 3 cultivar were induced under salt-stress but at non-significant level, relative to the untreated control plants. This induction level was also lower than that recorded in the Nain Ordinaire leaves. Treating the salt-stressed Elatius 3 plants with ALA significantly induced the expression of the three antioxidant genes, compared to the plants stressed with NaCl alone (Figure 7). Moreover, priming the non-stressed Elatius 3 plants with ALA significantly induced the expression of the three antioxidant genes, relative to the untreated control plants.

The aforementioned results indicate that the Nain Ordinaire cultivar tolerated salinity more than Elatius 3. This reflects that the Nain Ordinaire cultivar (comprising the highest variability level) is moderately tolerant to salinity, and that Elatius 3 (comprising the lowest variability level) is sensitive to salinity, suggesting that there is a sort of correlation between their genetic diversity level and salt tolerance degree. The results also confirmed the high potential of ALA to further enhance salt tolerance in these two pea genotypes by modulating the biosynthesis pathway of the photosynthetic pigments and up-regulating the antioxidant genes. ALA has an effective role against the adverse impacts induced by different plant environmental stresses. For instance, the foliar spray of ALA mitigated the membrane peroxidation and net photosynthetic rate inhibition induced by salt-stress in creeping bentgrass [38]. Furthermore, the exogenous ALA enhanced salt tolerance in tomato [39], swiss chard [40], rice [41], cucumber [42], and sicklepod [43]. As a result of its key role in the chlorophyll biosynthetic pathway, ALA promoted photosynthesis under numerous abiotic factors. Exogenous ALA enhanced the lettuce chlorophyll content reduced by UV-B stress [44]. Additionally, the foliar spray of ALA induced the chlorophyll fluorescence indexes in oilseed rape subjected to drought stress [45]. ALA promoted the gas exchange parameters in cauliflower under chromium stress [46]. Moreover, ALA induced the expression of antioxidant genes and genes encoding enzymes involved in Calvin cycle of photosynthesis in oilseed rape subjected to drought [47].

In conclusion, the present study revealed that the fatty acid profiling and AFLPs are efficient approaches to study the diversity analysis of the French pea germplasm. To our best knowledge, this investigation is among the first studies that have conducted marker-trait associations in pea. The results revealed a sort of correlation between the genetic diversity level and salt tolerance degree in pea genotypes. ALA conferred an enhanced tolerance to salinity in the French pea genotypes. Taken together, the results of this study might be used to improve pea crop. 

## 3. Materials and Methods

### 3.1. Plant Material

Twenty-five varieties of French pea (*Pisum sativum* L.) were used in this study (Table 1). Out of them, 19 cultivars were deposited at the Centre for Genetic Resources in the Netherlands, and six wild accessions (Elatius 1, Elatius 2, Elatius 3, Arvense 1, Arvense 2, and Arvense 3) were obtained from the French seed markets.

### 3.2. Pea Oil Content Estimation and Fatty Acid Analysis

Crude oil content estimation and fatty acid analysis of each of the 25 *Pisum sativum* L. accessions were carried out, as previously explained by Murcia and Rincon [36,37].

### 3.3. Molecular Diversity Analysis

#### 3.3.1. DNA Extraction and AFLP Analysis

The DNA was extracted from leaves of three individuals of each accession using a DNeasy Plant Kit (Qiagen, Paris, France), following the manufacturers’ procedures. The DNA samples were then subjected to AFLP analysis. Fifteen AFLP primer sets were chosen from the literature [30,35] and tested for polymorphism. Out of them, 12 primer sets exhibited a higher variability and were utilised to study the 25 accessions (Table 2). The AFLP analysis was conducted as explained by Vos et al. [48] with minor modifications, as reported in Simioniuc et al. [30]. The 12 AFLP primer pairs included E-ACA/M- CGA, E-ACA/M-CTG, E-AAC/M-CAA, E-ATG/M-CAA, E-AAA/M-CAC, E-AAC/M-CAC, E-ACA/M-CAC, E-ATC/M-CAC, E-ACA/M-CAG, E-ATG/M-CAG, E-AAT/M-CAT, and E-ATC/M-CAT (Table 2). The amplified products were then fragmented on 8% polyacrylamide gel. The gels were then stained using SYBR Gold stain and photographed (Sigma, Paris, France).

#### 3.3.2. AFLP Data Analysis

The AFLP bands were scored as present (1) and absent (0). PowerMarker 3.25 [49] and GenAlEx 6.5 [50] were used to measure the genetic diversity indices, including polymorphism percentage, polymorphic information content (*PIC*), the observed heterozygosity (*H_o_*), the fixation index (F), gene diversity, and the expected heterozygosity (*H_e_*). The cluster analysis was created using Nei’s distance [51].

### 3.4. Marker–Trait Association

The mixed linear model [52] was utilized for the marker–trait association analysis (AFLP data and crude oil content data). This analysis was carried out using TASSEL 3.0 [53]. A significance threshold for association analysis was set at *p* = 0.05.

### 3.5. Salt Tolerance Test and The Effect of 5-Aminolevulinic Foliar Spray in Pea

#### 3.5.1. Growth Conditions and Treatments

The seeds of the two pea cultivars (Nain Ordinaire and Elatius 3) were surface-sterilized in 8% NaClO for 5 min, rinsed thoroughly with sterile water, and allowed to germinate on filter paper saturated with sterile water for four days. The four-day old uniform pea plants were then transplanted into pots filled with soil consisting of sand, perlite, and peat (1:1:1, *v*/*v*/*v*). The pots were then sorted in a completely randomized design, with five replicates maintained under the controlled conditions of 26/21 °C day/night temperature, 16/8 h day/night photoperiod, and 80% humidity. The transplanted plants were watered every three days with a Hoagland solution comprising 0 and 150 mM NaCl over the experimental period (four weeks). A foliar spray of 5-aminolevulinic acid (6 ppm) was applied twice a week to the pea plants, and the untreated pea plants represented controls. After four weeks of plant transplantation, the plant samples were collected and utilized for further analysis.

#### 3.5.2. Determination of Contents of Photosynthetic Pigments

Contents of chlorophyll a (chl a) a, chlorophyll b (chl b), total chlorophyll (total chl), and carotenoids in the pea leaves were estimated following the methodology reported by Lichtenthaler and Wellburn [54].

#### 3.5.3. Antioxidant Enzymes Assay

The activities of the antioxidant enzymes, including peroxidase (POD), ascorbate peroxidase (APX), and catalase (CAT), were determined in the pea leaves following the method of Zhang and Kirkham [55].

#### 3.5.4. Gene Expression Analysis

Quantitative real-time PCR (qRT-PCR) analysis was assayed to study the expression of three antioxidant genes (*APX*, *CAT*, and *POD*) mediating the salt tolerance in pea. The total RNA was isolated from the pea leaves using a RNeasy Plant Mini kit, and the cDNA was synthesized using a Reverse Transcription kit (Qiagen). The qRT-PCR was then assayed using the QuantiTect SYBR Green PCR kit (Qiagen, Paris, France). The amplification conditions were set up as follows: 95 °C for 10 min; 35 cycles of 94 °C for 20 s, 62 °C for 30 s, 72 °C for 1 min; and 72 °C for 4 min. Gene specific-primers were used for amplification [56]. A melting-curve analysis was applied to verify the amplification specificity. *Actin* was served as a housekeeping gene. The relative expression levels were then estimated using the 2^−ΔΔ*C*t^ method.

#### 3.5.5. Statistical Analysis

One-way analysis of variance (ANOVA) and Duncan’s multiple range test were conducted. Data are means ± SE (*n* = 4), and values at *p* ≤ 0.05 differ significantly.

## Figures and Tables

**Table 1:** Composition of fatty acid of the 25 accessions of French pea.

Cultivar/Variety	Palmitic (C16:0)	Stearic (C18:0)	Oleic (C18:1)	Linoleic (C18:2)	Linolenic (C18:3)
Censeur	15.8	14.7	23.2	33.4	12.4
Centurion	16.7	15.8	23.3	33.2	12.2
Chemin Long	17.1	16.2	24.1	35.2	13.4
Chenille Mouchette	15.9	14.7	24.4	33.9	12.8
Clamart Nain Hatif	14.8	14.3	24.2	34.1	13.2
Electricite	16.1	15.7	23.2	33.4	12.7
Gali	17.0	16.1	22.8	32.1	14.1
Gloire de Quimper	18.2	14.8	24.3	32.8	13.2
Kalife	15.8	15.1	22.4	31.6	11.8
Landrieu Fascie	16.2	13.6	25.6	30.2	13.1
Myzar	18.4	15.2	23.7	32.8	13.3
Nain Couturier	16.4	15.0	22.8	31.7	12.7
Nain Ordinaire	16.1	14.9	24.1	33.2	12.2
Orfeo	15.6	14.7	22.8	33.6	13.4
Piver	16.8	15.8	23.3	32.4	13.6
Rex	14.9	13.9	20.6	29.8	14.0
Sachs Progress	16.3	15.1	21.8	31.1	13.9
Souchet	15.4	14.5	20.2	28.4	14.2
Starcovert	16.6	15.3	23.4	31.2	12.6
Elatius 1	14.0	13.1	19.2	27.4	11.2
Elatius 2	14.2	12.8	18.7	26.2	11.0
Elatius 3	13.8	13.5	20.1	25.9	11.9
Arvense 1	14.4	14.1	20.8	26.1	12.2
Arvense 2	13.6	13.2	19.4	25.8	11.7
Arvense 3	14.6	15.0	21.3	24.8	12.4

**Table 2:** AFLP primers sets, number of amplified fragments and diversity indices in pea accessions.

AFLP Primer Sets	No. of Fragments	No. of Polymorphic Fragments	Polymorphism (%)	Gene Diversity	PIC
E-ACA/M-CGA	86	54	63.8	0.52	0.47
E-ACA/M-CTG	89	51	57.3	0.58	0.54
E-AAC/M-CAA	51	35	68.6	0.43	0.38
E-ATG/M-CAA	56	33	58.9	0.39	0.36
E-AAA/M-CAC	71	47	66.2	0.51	0.47
E-AAC/M-CAC	37	23	62.2	0.38	0.34
E-ACA/M-CAC	51	41	80.4	0.49	0.44
E-ATC/M-CAC	62	44	70.9	0.37	0.33
E-ACA/M-CAG	41	21	51.2	0.28	0.23
E-ATG/M-CAG	31	19	61.3	0.21	0.16
E-AAT/M-CAT	29	15	51.7	0.35	0.31
E-ATC/M-CAT	65	40	61.5	0.41	0.37
Mean	55.75	35.25	62.83	0.41	0.37

E—5′-GACTGCGTACCAATTC-3′; M—5′-GATGAGTCCTGAGTAA-3′; PIC—polymorphic information content.

**Table 3:** Genetic diversity indices of the 25 varieties of *Pisum sativum* L.

Cultivar/Variety	*H*_o_	*H*_e_	*F*	PIC
Censeur	0.291	0.425	0.371	0.391
Centurion	0.312	0.392	0.244	0.352
Chemin Long	0.321	0.388	0.187	0.344
Chenille Mouchette	0.197	0.311	0.351	0.281
Clamart Nain Hatif	0.221	0.359	0.389	0.315
Electricite	0.198	0.291	0.304	0.262
Gali	0.258	0.321	0.211	0.282
Gloire de Quimper	0.321	0.424	0.302	0.374
Kalife	0.294	0.356	0.182	0.311
Landrieu Fascie	0.281	0.399	0.332	0.355
Myzar	0.265	0.317	0.151	0.272
Nain Couturier	0.274	0.392	0.361	0.348
Nain Ordinaire	0.311	0.477	0.421	0.416
Orfeo	0.292	0.411	0.362	0.372
Piver	0.231	0.397	0.441	0.352
Rex	0.262	0.225	−0.124	0.188
Sachs Progress	0.254	0.413	0.458	0.372
Souchet	0.199	0.314	0.361	0.272
Starcovert	0.311	0.282	−0.102	0.244
Elatius 1	0.281	0.391	0.331	0.344
Elatius 2	0.313	0.262	−0.152	0.238
Elatius 3	0.224	0.212	−0.039	0.184
Arvense 1	0.314	0.427	0.334	0.387
Arvense 2	0.322	0.462	0.421	0.411
Arvense 3	0.278	0.401	0.397	0.362
Mean	0.273	0.362	0.260	0.321

**Table 4:** Contents of chlorophyll a (Chl a), chlorophyll b (Chl b), total chlorophyll, and carotenoid in the leaves of the Nain Ordinaire cultivar, following salinity (150 mM NaCl) and 5-aminolevulinic acid ([ALA], 6 ppm) treatments.

NaCl Treatment	ALA (6 ppm)	Carotenoid (mg g^−1^ FW)	Chl a (mg g^−1^ FW)	Chl b (mg g^−1^ FW)	Total Chl (mg g^−1^ FW)
0 mM	−ALA	0.28 ± 0.09 ^c^	2.27 ± 0.14 ^c^	1.21 ± 0.08 ^c^	3.48 ± 0.11 ^c^
	+ALA	0.38 ± 0.06 ^a^	2.46 ± 0.18 ^a^	1.36 ± 0.05 ^a^	3.82 ± 0.12 ^a^
150 mM	−ALA	0.25 ± 0.08 ^c^	2.21 ± 0.14 ^c^	1.16 ± 0.07 ^c^	3.37 ± 0.15 ^c^
	+ALA	0.31 ± 0.09 ^b^	2.29 ± 0.11 ^b^	1.28 ± 0.05 ^b^	3.57 ± 0.13 ^b^

Data are means ± standard error (SE) (*n* = 4). Different letters next to the numbers in the same column indicate significant differences between treatments (*p* ≤ 0.05).

**Table 5:** Contents of chlorophyll and carotenoid in the leaves of the Elatius 3 cultivar following salinity (150 mM NaCl) and 5-aminolevulinic acid ([ALA], 6 ppm) treatments.

NaCl Treatment	ALA (6 ppm)	Carotenoid (mg g^−1^ FW)	Chl a (mg g^−1^ FW)	Chl b (mg g^−1^ FW)	Total Chl (mg g^−1^ FW)
0 mM	−ALA	0.26 ± 0.05 ^b^	2.18 ± 0.12 ^b^	1.16 ± 0.07 ^b^	3.34 ± 0.10 ^b^
	+ALA	0.31 ± 0.06 ^a^	2.42 ± 0.11 ^a^	1.52 ± 0.09 ^a^	3.94 ± 0.15 ^a^
150 mM	−ALA	0.18 ± 0.07 ^c^	1.72 ± 0.12 ^d^	0.94 ± 0.04 ^d^	2.66 ± 0.12 ^c^
	+ ALA	0.25 ± 0.05 ^b^	1.95 ± 0.10 ^c^	1.13 ± 0.07 ^c^	2.08 ± 0.11 ^d^

Data are means ± SE (*n* = 4). Different letters next to the numbers in the same column indicate significant differences between treatments (*p* ≤ 0.05).
